# Can the Market Recognize the Value of the Corporate Governance Mechanism of Chinese Listed Companies?—Empirical Evidence From COVID-19

**DOI:** 10.3389/fpubh.2021.812253

**Published:** 2022-01-26

**Authors:** Jianwei Li, Yunbiao Ma, Beibei Shi, Yi Yang

**Affiliations:** ^1^School of International Trade and Economics, University of International Business and Economics, Beijing, China; ^2^School of Finance, Beijing College of Finance and Commerce, Beijing, China; ^3^School of Accounting, Central University of Finance and Economics, Beijing, China

**Keywords:** corporate governance, COVID-19, stock price, ownership structure, board structure, managerial compensation

## Abstract

This paper studies whether the market can recognize the value of corporate governance mechanisms (ownership structure, board structure, and managerial incentives) of Chinese listed companies. We find that when companies are faced with “black swan” events, such as COVID-19, non-state-owned enterprise are found to be more valuable, that is, the stock price of non-state-owned enterprises are more immune to the negative shocks of COVID-19. For board structure, the arrangement of the duality of chairman and CEO is found to be more valuable and can effectively alleviate the negative shocks of the epidemic on the stock price. For managerial incentives mechanisms, it shows that management shareholding, management compensation, and executive stock options are all effective mechanisms and can better withstand the negative shocks of the COVID-19 epidemic on the stock price of companies. This paper sheds light on the value of corporate governance mechanisms in the Chinese capital market from the perspective of investors, which enriches literature in the field of corporate governance.

## Introduction

The Coronavirus Disease-2019 (COVID-19) epidemic in 2020 as the “black swan” exogenous event not only severely endangered the lives and health of the people but also had a serious impact on the capital market. It has caused widespread negative sentiment and led to market turbulence, harming the interests of investors and aggravating the instability of the capital market. In this case, which kind of corporate governance mechanisms are more effective? That is, which kind of corporate governance mechanisms make some companies more “immune” to the COVID-19 shock than others? So far, this issue is still not well-studied.

In this paper, we have examined the relationship between corporate governance mechanisms and stock price reactions to the COVID-19 pandemic using Chinese data as the COVID-19 first broke out in China, which offers a totally exogenous event to establish a clear causal relation between corporate governance characteristics and stock price reactions to the COVID-19 pandemic. Using theories of corporate governance to frame our study and data on over 3,000 Chinese listed firms during the first two quarters of 2020, we consider three important aspects of corporate governance mechanisms: (1) ownership structure, such as ownership concentration, multiple blockholders, and nature of ownership, (2) board structure, such as board size, board independence, board gender diversity, and the duality of Chairman and CEO, (3) managerial incentives, such as managerial ownership, managerial compensation, and executive stock options.

Based on a sample of Chinese listed firms, this paper finds that the pandemic-induced drop in stock prices was milder among firms with (a) non-SOEs, (b) Chairman and CEO duality, (c) higher managerial compensation, but ownership concentration, multiple blockholders, the board size, board independence, and board gender diversity have no significant effect on mitigating the negative impact of the epidemic on stock prices.

This paper contributes to the scant literature by shedding empirical light on the value of corporate governance mechanisms in the Chinese capital market from the perspective of investors. Although existing studies have conducted in-depth discussion on the role of corporate governance mechanisms in China's capital market, they mainly focus on the impact of corporate governance mechanisms on the company's decision-making and behaviors, few studies pay attention to whether the market can recognize the value of corporate governance mechanism of Chinese listed firms. This paper helps to fill in this gap. Moreover, existing works of literature have not reached a consensus on the role of internal corporate governance mechanisms, which is mainly due to the problem of endogeneity. Using COVID-19 as an exogenous shock helps us find a causal relationship between corporate governance mechanisms and firm value.

## Theoretical Background

Corporate governance is a series of institutional arrangements to reduce corporate agency problems, so as to ensure that investors can get returns from their investments. As China is moving away from a planned economy to a market-oriented one, more and more scholars pay attention to the effectiveness of China's corporate governance mechanisms. The most important internal corporate governance mechanisms include ownership structure, board structure, and manager incentive mechanism. Existing literature has formed different theories and views on these corporate governance mechanisms of Chinese listed companies through theoretical and empirical research.

First, *ownership structure*. *Concentrated ownership* or large shareholders within firms is the most important governance feature of Chinese listed companies (1). Alignment effects encourage large shareholders to manage the company or monitor managers more actively. On the other hand, the existence of large shareholders can also bring additional costs by tunneling the listed companies. In face of COVID-19, whether large shareholders help companies tide over the difficulties or conspiracy to infringe on the interests of small investors, needs to be empirically tested.

A growing literature proposes that relative to diffuse ownership and concentrated ownership, an optimal ownership structure may be to have *multiple blockholders*. These multiple blockholders provide oversight to the firms and also over each other, which not only minimizes the agency cost between owners and managers, but also the agency cost between blockholders and small investors (2, 3). However, multiple blockholders may also conspire to infringe on the interests of minority shareholders. Moreover, major shareholders can form a control alliance with the least cash flow right through collusion, so as to have a greater incentive to carry out more predatory behavior (4, 5). Therefore, whether the existence of multiple blockholders represents an optimal ownership structure in the COVID-19 epidemic is an empirical question.

The *nature of ownership* in Chinese listed companies plays a key role in terms of governance. State-owned enterprises (SOEs) pursue political objectives rather than maximizing profit, such as investing in projects that are beneficial to society but not cost-effective to the companies, thus damaging the value of the company. In addition, there are often complex principal-agent relationships in SOEs. This complex principal-agent relationship not only causes the problem of insufficient incentive in SOEs but also leads to the problem of weak supervision in SOEs However, private enterprises are more profit-oriented and have more perfect manager market and profit-oriented supervisors, so they have comparative advantages in supervision (6, 7). On the other hand, SOEs are less restricted by government policies, have easier access to monopolistic industries, and are more likely to receive subsidies from the government when they are in difficulties. It can be seen that SOEs and private enterprises have their own advantages and disadvantages in the face of “black swan” incidents, such as the COVID-19 epidemic.

Second, *board structure*. The board of directors perform the critical function of monitoring and advising the managers. A strand of literature suggests that a greater level of board size, board independence, board gender diversity, and the separation of Chairman and CEO allow for more effective monitoring and provide more valuable advice (8–10). However, with the changes in the internal and external environment of the company and the emergence of large multinational corporations, the above views began to be questioned. Based on the principal-agent theory, scholars believe that too large board size can not only promote the growth of enterprise performance, but will also reduce the efficiency of the company because of high coordination costs and organizational costs (11, 12). Therefore, which kind of board structure can help firms withstand the negative impact of COVID-19 on stock price requires an empirical test.

Third, *managerial compensation*. The optimal contract theory believes that pay arrangements aim to maximize shareholders' value and a higher level of manager compensation, such as shareholding, cash compensation, and executive options, can alleviate the agency problems between managers and shareholders (13). However, the entrenchment theory believes that manager compensation contract deviates from optimal contracting as directors are often captured or subject to influence by managers, and a higher level of manager compensation provides suboptimal incentives and thereby damage shareholders' value (14–16). During the COVID-19 pandemic, whether managerial compensation is more consistent with optimal contract theory or the entrenchment theory, and whether it can alleviate the impact of COVID-19 on stock prices are empirical questions.

## Data, Variables, and Methodology

Our sample consists of all Chinese A share listed companies between January 20 and April 8 in 2020. The reason for choosing this special research interval is that On January 20, 2020, Hubei Province initiated a secondary response to public emergencies and Wuhan was locked down on January 23, indicating that the epidemic broke out, and Wuhan was unblocked on April 8, indicating that the epidemic was basically controlled. The financial and the confirmed cases of COVID-19 data are from the CSMAR database. Following extant literature, we exclude financial services firms and firm-year observations with an incomplete financial date for the control variables. Continuous variables are winsorized at 1% in each tail.

To study the effect of corporate governance on stock price during the period of the COVID-19 pandemic, we follow Ding et al. (17) and use the following regression model:


$$\begin{array}{l} Ret_{i,t}=\alpha_{0}+\beta_{1}\times COVID_{p,t}+\beta_{2}\times G_{i,pre2020}\times \\ COVID_{p,t}+\beta_{n}\times F_{i,pre2020}\times COVID_{p,t}+\mu_{i}+\mu_{t}+\varepsilon_{i,t} \end{array}$$

Where *i* refers to the individual of the company, p refers to the city where the company is registered, and t refers to the number of weeks. *Ret*_*i,t*_ is the weekly stock return of company i from the last trading day of week *t*−1 to the last trading day of week *t*. *COVID19*_*p,t*_ is the growth rate of the number of confirmed cases of COVID-19 over week *t* in province *p* as follows.


$$\begin{array}{l} {COVID}_{p,t}=Ln\left(1+Confirmed\;Cases_{p,t}\right)-\\ Ln\left(1+Confirmed\;Cases_{p,t-1}\right) \end{array}$$

*Confirmed Cases*_*p,t*_ represents the cumulative number of confirmed cases in province *p* as of Friday in week *t*.

*G*_*i,pre*2020_ is corporate governance characteristics, such as ownership structure, board structure, and managerial compensation.

We consider three characteristics of ownership structure: (1) *SOE* equals 1 if controlling shareholders is state-owned, and zero otherwise. (2) *Top1* equals the percentage of shareholdings of controlling shareholders in total shares. (3) *Multiple* equals 1 if there are two or more shareholders holding more than 10% of the shares and zero otherwise.

We examine four measures of board structure: (1) *BoardSize is* the logarithm of numbers of board members. (2) *IndepRatio is* the percentage of independent directors in the board of directors. (3) *FemaleRatio is* the percentage of female directors in the board of directors. (4) *Dual* equals 1 if the chairman of the board serving as CEO and zero otherwise.

We consider three features of managerial compensation: (1) *Msh* equals the percentage of shareholdings of managers in total shares. (2) *CashPay* equals the logarithm of management cash compensation. (3) *StockPay* equals 1 if managers have a stock option, and zero otherwise.

*F*_*i,pre*2020_ is control variable, such as *Size* (natural logarithm of the book value of total assets), *Roa* (ratio of net profit to total assets), *Lev* (debt-to-asset ratio), and *Cash* (the total amount of cash holding divided by total assets). We also include firm and week dummies. The SEs are clustered at the firm level. β_1_ captures the effect of COVID-19 on the stock price. β_2_ captures the effect of corporate governance on stock price during the period of the COVID-19 pandemic.

Table 1 presents descriptive statistics on the main variables of this paper. Among them, the average weekly rate of return (Ret) of the sample companies is 0.03%. Its minimum value is −17.54%, and the maximum value is 25.69%. For the confirmed cases of COVID19, the average weekly increase (COVID19) was 18.4%, and the maximum increase was 368.89%. The SOEs accounted for 29.5% of the total sample. The average shareholding ratio of the largest shareholder is 33.08%, the descriptive statistical results of other variables will not be repeated here.

**Table 1:** Descriptive statistics.

	***N***	**Mean**	**Sd**	**Min**	**Max**
Ret	37,621	0.0003	0.0821	−0.1754	0.2569
COVID-19	2,563	0.1840	0.4601	0.0000	3.6889
**Firm characteristics**					
SOE	37,621	0.2950	0.4560	0.0000	1.0000
Top1	37,621	0.3308	0.1453	0.0899	0.7272
Multiple	37,621	0.2591	0.4381	0.0000	1.0000
BoardSize	37,621	2.1048	0.2002	1.6094	2.6391
Dual	37,621	0.3003	0.4584	0.0000	1.0000
IndepRatio	37,621	0.3793	0.0543	0.3077	0.5714
FemaleRatio	37,621	0.1601	0.1260	0.0000	0.5000
Msh	37,621	0.1454	0.1978	0.0000	0.6858
CashPay	37,621	13.5913	0.6626	12.2094	15.5542
StockPay	37,621	0.3331	0.4713	0.0000	1.0000
Size	37,621	22.3487	1.3813	19.7531	26.5744
Roa	37,621	0.0194	0.1173	−0.6803	0.2074
Lev	37,621	0.1632	0.1491	0.0000	0.6115
Cash	37,621	0.1667	0.1171	0.0112	0.5764

## Empirical Results

### Ownership Structure

Table 2 presents the estimation results for the effect of ownership structure on stock price sensitivity to COVID-19. As a preliminary benchmark, we simply assess the relationship between stock returns and economies' exposure to the COVID-19 pandemic. The result in column (1) of Table 2 shows that the coefficients of COVID19 are significantly negative, suggesting that exposure of a province to the pandemic has a negative impact on the stock market performance of firms in that province.

**Table 2:** Ownership structure and stock price reactions.

	**(1)**	**(2)**	**(3)**	**(4)**	**(5)**
COVID-19	−0.0041^***^	−0.0562^***^	−0.0467^***^	−0.0467^***^	−0.0565^***^
	(−3.47)	(−7.59)	(−6.61)	(−6.71)	(−7.62)
SOE^*^COVID-19		−0.0037^***^			−0.0037^***^
		(−3.86)			(−3.72)
Top1^*^COVID-19			−0.0024		−0.0001
			(−0.73)		(−0.04)
Multiple^*^COVID-19				0.0006	0.0004
				(0.57)	(0.44)
Size^*^COVID-19		0.0024^***^	0.0020^***^	0.0019^***^	0.0024^***^
		(7.21)	(5.99)	(6.25)	(7.03)
Roa^*^COVID-19		0.0239^***^	0.0244^***^	0.0241^***^	0.0240^***^
		(5.90)	(5.89)	(5.86)	(5.86)
Lev^*^COVID-19		−0.0042	−0.0047	−0.0046	−0.0043
		(−1.24)	(−1.38)	(−1.35)	(−1.25)
Cash^*^COVID-19		−0.0001	−0.0006	−0.0009	−0.0001
		(−0.02)	(−0.16)	(−0.22)	(−0.02)
Firm effects	Yes	Yes	Yes	Yes	Yes
Week effects	Yes	Yes	Yes	Yes	Yes
N	37,621	37,621	37,621	37,621	37,621
R^2^_Adjusted	0.411	0.412	0.412	0.412	0.412

*The t-statistics reported in parentheses are based on robust SEs clustered by firm. The t-statistics are in parentheses*.

***, **, and * *denote statistical significance at the 1, 5, and 10% level, respectively*.

We then assess the differential sensitivity of stock price reactions to COVID-19 as a function of firms' pre-existing levels of ownership structure. The results in column (2) of Table 2 show that the coefficient of interactive item between SOE and COVID-19 is significantly negative, indicating that firms controlled by the government tend to experience more stock price declines during the COVID-19 crisis. This may be because SOEs take more social responsibilities, such as solving unemployment issues and making more donations when facing economic stagnation caused by the COVID-19 epidemic. These social responsibilities will further increase the economic burden of SOEs, thereby triggering investors to sell the stocks of SOEs. However, the result in columns (3) and (4) of Table 2 indicates that ownership concentration and multiple blockholders do not exhibit any significant role in resisting the negative impact of the COVID-19 epidemic on the company's stock price. In column (5) of Table 2, we simultaneously examine all three pre-2020 ownership structure characteristics. Each of the indicators enters statistically significantly, with the same sign and similar estimated coefficient as the earlier findings.

### Board Structure

We test whether board structure characteristics could alleviate the impact of the COVID-19 epidemic on the corporate stock prices. Table 3 presents the results. Columns (4) and (5) of Table 3 show that the coefficient of interactive item between the duality of chairman and CEO and COVID-19 is significantly positive, indicating that the duality of chairman and CEO can help. This conclusion supports the concept of “Stewardship Theory”, that is, when the CEO serves as chairman of the company, the CEO has the absolute leadership of the company, can more effectively make decisions, and allocates resources to withstand the negative impact of the COVID-19 epidemic. Therefore, investors respond better to this mechanism. However, the coefficients of interactions between other board structure characteristics and COVID-19 are not significant, indicating that these characteristics are not effective in resisting the negative impact of the COVID-19 epidemic.

**Table 3:** Board structure and stock returns in response to COVID-19.

	**(1)**	**(2)**	**(3)**	**(4)**	**(5)**
COVID-19	−0.0441^***^	−0.0517^***^	−0.0463^***^	−0.0480^***^	−0.0474^***^
	(−5.81)	(−7.56)	(−6.50)	(−6.61)	(−5.10)
BoardSizen^*^COVID-19	−0.0018				−0.0022
	(−0.75)				(−0.72)
IndepRatio^*^COVID-19		0.0001			−0.0083
		(0.02)			(−0.86)
FemaleRatio^*^COVID-19			0.0038		0.0032
			(1.07)		(0.90)
Dual^*^COVID-19				0.0032^***^	0.0032^***^
				(3.15)	(3.11)
Size^*^COVID-19	0.0020^***^	0.0021^***^	0.0019^***^	0.0020^***^	0.0023^***^
	(6.19)	(6.98)	(6.22)	(6.23)	(6.61)
Roa^*^COVID-19	0.0241^***^	0.0241^***^	0.0240^***^	0.0239^***^	0.0241^***^
	(5.87)	(5.84)	(5.85)	(5.85)	(5.85)
Lev^*^COVID-19	−0.0045	−0.0045	−0.0046	−0.0047	−0.0045
	(−1.32)	(−1.31)	(−1.34)	(−1.37)	(−1.31)
Cash^*^COVID-19	−0.0008	−0.0011	−0.0009	−0.0008	−0.0010
	(−0.20)	(−0.27)	(−0.22)	(−0.21)	(−0.26)
Firm effects	Yes	Yes	Yes	Yes	Yes
Week effects	Yes	Yes	Yes	Yes	Yes
N	37,621	37,621	37,621	37,621	37,621
R^2^_Adjusted	0.412	0.412	0.412	0.412	0.412

*The t-statistics reported in parentheses are based on robust standard errors clustered by firm. The t-statistics are in parentheses*.

***, **, and * *denote statistical significance at the 1, 5, and 10% level, respectively*.

### Managerial Incentives

Similarly, we test whether managerial compensation contracts can resist the impact of the COVID-19 epidemic on the corporate stock prices. Table 4 shows that the interactions between COVID-19 and management ownership, management cash payment, and executive option are all significantly positive. These results indicate that managerial compensation contracts can help withstand the negative impact of the COVID-19 epidemic. This conclusion supports the “optimal contract theory” of managerial compensation, that is, managerial compensation contracts alleviate the agency conflict between managers and shareholders, encouraging the management to maximize shareholders' wealth.

**Table 4:** Managerial compensation and stock returns in response to COVID-19.

	**(1)**	**(2)**	**(3)**	**(4)**
COVID-19	−0.0546^***^	−0.0827^***^	−0.0496^***^	−0.0883^***^
	(−7.64)	(−8.23)	(−7.02)	(−8.47)
Msh^*^COVID-19	0.0062^**^			0.0044^*^
	(2.45)			(1.65)
CashPay^*^COVID-19		0.0046^***^		0.0042^***^
		(5.45)		(4.96)
StockPay^*^COVID-19			0.0038^***^	0.0028^***^
			(4.12)	(2.90)
Size^*^COVID-19	0.0022^***^	0.0008^**^	0.0020^***^	0.0012^***^
	(7.09)	(2.20)	(6.43)	(3.25)
Roa^*^COVID-19	0.0224^***^	0.0222^***^	0.0227^***^	0.0203^***^
	(5.44)	(5.42)	(5.49)	(4.88)
Lev^*^COVID-19	−0.0043	−0.0025	−0.0042	−0.0022
	(−1.26)	(−0.74)	(−1.21)	(−0.64)
Cash^*^COVID-19	−0.0010	−0.0033	−0.0002	−0.0027
	(−0.26)	(−0.83)	(−0.04)	(−0.68)
Firm effects	Yes	Yes	Yes	Yes
Week effects	Yes	Yes	Yes	Yes
N	37,621	37,621	37,621	37,547
R^2^_Adjusted	0.412	0.412	0.412	0.413

*The t-statistics reported in parentheses are based on robust standard errors clustered by firm. The t-statistics are in parentheses*.

***, **, and * *denote statistical significance at the 1, 5, and 10% level, respectively*.

## Conclusion

Based on a sample of Chinese listed firms, this paper finds that non-SOEs and firms with Chairman and CEO duality and high managerial compensation can better withstand the impact of the epidemic on corporate stock price, but ownership concentration, multiple blockholders, the size of the board, the proportion of independent directors, and female directors have no significant effect on mitigating the negative impact of the epidemic on stock prices. This paper helps to understand the role of corporate governance characteristics in stock price reactions to COVID-19 in 2020. In addition, for listed companies, the research conclusions of this paper have important enlightenment significance for them to formulate an effective corporate governance mechanism to reduce the impact of the “black swan” phenomenon in the capital market.

## Data Availability Statement

The original contributions presented in the study are included in the article/supplementary material, further inquiries can be directed to the corresponding author/s.

## Author Contributions

JL contributed to empirical analyses and writing the paper. YM contributed to data collection and writing the paper. BS contributed to methodology and writing the paper. YY contributed to supervision and paper writing. All authors contributed to the article and approved the submitted version.

## Funding

The authors acknowledge the financial support from the National Social Science Foundation of China. Research on the Mechanism and Path of Precise Financial Support to Enhance the Resilience of Private Economy, Grant No. 19BJL059.

## Conflict of Interest

The authors declare that the research was conducted in the absence of any commercial or financial relationships that could be construed as a potential conflict of interest.

## Publisher's Note

All claims expressed in this article are solely those of the authors and do not necessarily represent those of their affiliated organizations, or those of the publisher, the editors and the reviewers. Any product that may be evaluated in this article, or claim that may be made by its manufacturer, is not guaranteed or endorsed by the publisher.
